# Supplementary material for: Content-rich biological network constructed by mining PubMed abstracts
Source: BMC Bioinformatics. 2004 Oct 8;5:147. doi: 10.1186/1471-2105-5-147 (PMC528731; doi:10.1186/1471-2105-5-147)
Supplement: Additional File 2 — The original results of the above study (non-essential files are deleted to keep the file size under the limit set by BMC bioinformatics). [file 1471-2105-5-147-S2.bz2 › chilibotAdditionalFile2/dip40/26ID8999548E89/html/IFNG_IL18.html]

 


 **IFNG** and **IL18** 
  
Found 772 abstracts in PubMed, retrieved 40.  
 

 What does Google say? 
 PDF only 
| .edu only 

---

**Interactive relationship** (e.g. stimulation, inhibition, etc)

**Inhibitory relationship**- Anti IL 12 and anti IL 18  [ **IL18** ]  antibodies decreased IFN gamma  [ **IFNG** ]  production by these cells.  Ref: 12857673 Am J Physiol Lung Cell Mol PhysiolAm J Physiol Lung Cell Mol Physiol,
- IL 18  [ **IL18** ]  translational inhibition restricts IFN gamma  [ **IFNG** ]  expression in crescentic glomerulonephritis.  Ref: 12787406 Kidney Int, 2003
- IL 18  [ **IL18** ]  translational inhibition restricts IFN gamma  [ **IFNG** ]  expression in crescentic glomerulonephritis.  Ref: 12787406 Kidney Int, 2003
- IL 18  [ **IL18** ]  mRNA levels were reduced p=0.019 and the IL 4 Interferon gamma  [ **IFNG** ]  ratio elevated p=0.01 in stimulated peripheral blood mononuclear cells from infants with acute bronchiolitis.  Ref: 12773328 Am J Respir Crit Care Med< MedlineTA>Am J Respir Crit Care Med, 2003
- This bias was dependent on keratinocyte derived interferon alpha beta and interleukin 18  [ **IL18** ] , as neutralization of both interferon alpha beta and interleukin 18  [ **IL18** ]  in the keratinocyte culture supernatant reduced the development of interferon gamma  [ **IFNG** ]  producing Th cells.  Ref: 12787126 J Invest Dermatol< MedlineTA>J Invest Dermatol, 2003
- Thus, blockade of IL 18  [ **IL18** ]  appears to be a viable clinical target to combat the pathologic consequences of sepsis via IFN gamma  [ **IFNG** ]  mechanisms.  Ref: 12792854 J Infect Dis, 2003
- RESULTS IL 12 IL 18  [ **IL18** ]  EGT inhibited tumor growth significantly better than IL 12 EGT, which was consistent with significantly higher intratumoral levels of IFN gamma  [ **IFNG** ] .  Ref: 12820368 Anticancer Res< MedlineTA>Anticancer Res, 2003
**Stimulatory relationship**- Nai ; ve CD4 T cells differentiate into interferon gamma  [ **IFNG** ]  IFN gamma  [ **IFNG** ]  producing Th1 effector cells when stimulated by interleukin 18  [ **IL18** ]  IL 18  [ **IL18** ]  and IL 12.  Ref: 12829194 Cardiovasc Res< MedlineTA>Cardiovasc Res, 2003
- Naive CD4 CD62L Tcells from normal C57BL 6 mice or from DO11.10 Rag2 OVA specific TCR transgenic mice secreted substantial amounts of IFN gamma  [ **IFNG** ]  when stimulated concurrently with high dose IL 2 plus IL 12 or IL 18  [ **IL18** ] .  Ref: 12811838 Eur J Immunol, 2003
- However, because interleukin IL 18  [ **IL18** ]  acts synergistically with IL 12 to augment Th1 responses and interferon IFN gamma  [ **IFNG** ] , we designed an EGT protocol using IL 12 IL 18  [ **IL18** ]  with the aim of enhancing the antitumor effects obtained with IL 12 alone.  Ref: 12820368 Anticancer Res< MedlineTA>Anticancer Res, 2003
- Our data show that enhanced IFN gamma  [ **IFNG** ]  production in the lungs of bleomycin treated mice is at least partly IL 12 and IL 18  [ **IL18** ]  dependent.  Ref: 12857673 Am J Physiol Lung Cell Mol PhysiolAm J Physiol Lung Cell Mol Physiol, 2003
- Injection of recombinant active IL 18  [ **IL18** ]  in WKY rats with anti GBM GN was associated with an increase of glomerular IFN gamma  [ **IFNG** ]  levels, proliferating cell nuclear antigen PCNA ED1 cells, and PCNA CD8 cells, with worsening of glomerular injury.  Ref: 12787406 Kidney Int, 2003
- IL 18  [ **IL18** ]  is a cytokine structurally and functionally related to IL 1 that, in synergy with IL 12, stimulates the synthesis of IFN gamma  [ **IFNG** ]  from T lymphocytes and natural killer cells.  Ref: 12884303 Eur J ImmunolEur J Immunol, 2003
- However, the IFN gamma  [ **IFNG** ]  IL 18  [ **IL18** ]  ratio was significantly lower in patients with an APACHE II > =10, and in those with culture positive peritonitis, as compared with the other patients.Peritoneal IL 18  [ **IL18** ]  concentration increased in response to intraperitoneal bacterial infection and seemed to reflect the severity of peritonitis.  Ref: 12781884 Am J Surg, 2003
**Neutral relationship**- Interferon IFN gamma  [ **IFNG** ]  inducing factor was previously termed interleukin IL 18  [ **IL18** ] .  Ref: 12792854 J Infect Dis, 2003
- IL 18  [ **IL18** ]  activated the binding of NF kappaB to the IFN gamma  [ **IFNG** ]  promoter NF kappaB site.  Ref: 12759422 J Immunol, 2003
- IL 12 and IL 18  [ **IL18** ]  activate the synthesis of IFN gamma  [ **IFNG** ]  by innate immune cells, including NK, NKT and gammadeltaT cells and promote the differentiation of Th1 type acquired immune responses.  Ref: 12825993 Rev Iberoam Micol, 2002
- In fact, IL 18  [ **IL18** ]  was originally identified as an IFN gamma  [ **IFNG** ]  inducing factor, and IFN gamma  [ **IFNG** ]  production is the hallmark of the Th1 response.  Ref: 12784053 Blood Purif, 2003
- In the gut of non infected piglets, IL 1 beta, IL 8 and IL 18  [ **IL18** ]  were detected whereas TNF alpha and IFN gamma  [ **IFNG** ]  were mostly missing.  Ref: 12879755 Folia Microbiol (Praha), 2003
- Recombinant active IL 18  [ **IL18** ]  rIL 18 was used to further identify its effect on IFN gamma  [ **IFNG** ]  production during this GN.  Ref: 12787406 Kidney Int, 2003
- CD4 and CD8 T cells induced IL 18  [ **IL18** ]  from DC and primed for IL 12 p70 secretion via interferon gamma  [ **IFNG** ]  IFN gamma  [ **IFNG** ]  or tumour necrosis factor alpha TNF alpha.  Ref: 12871213 Immunology, 2003
- Interleukin 18  [ **IL18** ]  IL 18  [ **IL18** ]  can act together with IL 12 in promoting the generation of IFN gamma  [ **IFNG** ]  producing Th1 cells.  Ref: 12796406 Clin Cancer Res, 2003
- IL 18  [ **IL18** ]  or IL 21 alone induced a modest expression of the IFN gamma  [ **IFNG** ]  gene but a combination of IL 21 and IL 18  [ **IL18** ]  efficiently up regulated IFN gamma  [ **IFNG** ]  production.  Ref: 12759422 J Immunol, 2003
- Expression of the interleukin 23 IL 23 p19, IL 12p35, IL 18  [ **IL18** ] , interferon gamma  [ **IFNG** ]  IFN gamma  [ **IFNG** ]  and Mig monokine induced by IFN gamma  [ **IFNG** ]  genes was induced in the DCs that were cultured with A11 CD40L but not with A11 cells, and P40, the subunit of both IL 12 and IL 23, was secreted from the cocultured DCs.  Ref: 12768190 Cancer Gene Ther, 2003
- Interleukin IL 18  [ **IL18** ]  is a novel cytokine that has recently been characterized as an inducer of interferon gamma  [ **IFNG** ]  IFN gamma  [ **IFNG** ] .  Ref: 12781884 Am J Surg, 2003
- CONCLUSION These data suggest that the translational control of IL 18  [ **IL18** ]  expression by its 5 UTR limits the production of IL 18  [ **IL18** ] , resulting in restricted expression of mRNA and protein IFN gamma  [ **IFNG** ]  in this model of GN.  Ref: 12787406 Kidney Int, 2003
- The most salient biological property of IL 18  [ **IL18** ] , linking this cytokine to the Th1 response, is its ability to induce interferon gamma  [ **IFNG** ]  IFN gamma  [ **IFNG** ] .  Ref: 12784053 Blood Purif, 2003
- Interleukin 18  [ **IL18** ]  IL 18  [ **IL18** ] , a monokine that shares functional abilities with IL 12, is a potent inductor of interferon gamma  [ **IFNG** ]  IFN gamma  [ **IFNG** ]  by Th1 and natural killer NK cells.  Ref: 12858406 J Med Virol, 2003
- Moreover, IFN gamma  [ **IFNG** ]  mRNA steady state levels were correlated positively with those of IFNAR 1, IRF 1, and IL 18  [ **IL18** ] , suggesting a coordinated induction of these genes.  Ref: 12794720 J Med Virol< MedlineTA>J Med Virol, 2003
- The protective effect of IL 18  [ **IL18** ]  bp Fc was accompanied by modified ex vivo immune responses, in that spleen cells and peritoneal macrophages contained fewer IFN gamma  [ **IFNG** ]  secreting cells and released lower amounts of nitrite an index of nitric oxide production and IL 1beta.  Ref: 12884303 Eur J ImmunolEur J Immunol, 2003
- Moreover, they deliver additional support for the view that IFN gamma  [ **IFNG** ]  still has to be considered as a mediator that determines the outcome of inflammation, e.g., via its ability to activate IL 18  [ **IL18** ]  expressing cells and to initiate a delayed type hypersensitivity reaction.  Ref: 12794718 J Med Virol, 2003
- These results indicate that combined administration of IL 18  [ **IL18** ]  and low dose IL 2 could be a new model for cancer immunotherapy, which probably engages the activation of natural killer cells through interferon gamma  [ **IFNG** ]  and Fas L dependent pathways.  Ref: 12806277 J Immunother< MedlineTA>J Immunother, 2003

**Non-interactive relationship** (e.g. studied together, co-existance, homology, etc.)

- However, mRNA expression for IL 18  [ **IL18** ]  and interferon gamma  [ **IFNG** ]  IFN gamma  [ **IFNG** ]  was downregulated, and the expression of mRNA for the anti inflammatory cytokine transforming growth factor beta4 TGF beta 4 was upregulated.  Ref: 12859858 J Interferon Cytokine Res, 2003
- Expression of the chemokine IP 10 correlates with the accumulation of hepatic IFN gamma  [ **IFNG** ]  and IL 18  [ **IL18** ]  mRNA in chronic hepatitis C but not in hepatitis B.  Ref: 12794718 J Med Virol, 2003
- To investigate the intra hepatic activation of the IFN system in patients affected by chronic HCV infection in comparison with that observed in a non infectious liver disease such as non alcoholic steatohepatitis, we measured the liver steady state mRNA levels of interferon alpha, interferon beta and interferon gamma  [ **IFNG** ]  as well as of IFN related genes IFNAR 1, STAT1alpha, PKR, 2 5 AS, IRF 1, ICE and IL 18  [ **IL18** ] .  Ref: 12794720 J Med Virol< MedlineTA>J Med Virol, 2003
- T bet deficient DCs were significantly impaired in their capacity to secrete IFN gamma  [ **IFNG** ]  after both stimulation with IL 12 alone or in combination with IL 18  [ **IL18** ] .  Ref: 12802010 Proc Natl Acad Sci U S A< MedlineTA>Proc Natl Acad Sci U S A, 2003
- Now we have studied the effect of IL 15, IL 18  [ **IL18** ] , and IL 21 on IFN gamma  [ **IFNG** ]  gene expression in more detail in human NK and T cells.  Ref: 12759422 J Immunol, 2003
- Mice deficient in IFN gamma  [ **IFNG** ]  are not resistant to lethal endotoxemia but are resistant when treated with neutralizing antibodies to IL 18  [ **IL18** ]  and challenged with a lethal injection of some endotoxins.  Ref: 12792854 J Infect Dis, 2003
- However, high flux HD restores whole blood IFN gamma  [ **IFNG** ]  production without significant changes in free IL 18  [ **IL18** ] .  Ref: 12784048 Blood Purif, 2003
- the role of IL 18  [ **IL18** ]  in the production of IFN gamma  [ **IFNG** ]  during these pathologies has not been studied.  Ref: 12787406 Kidney Int, 2003
- Sepsis is initiated and perpetuated by the overzealous systemic production of proinflammatory cytokines such as tumor necrosis factor TNF alpha, interferon IFN gamma  [ **IFNG** ] , interleukin IL 12, and IL 18  [ **IL18** ]  sometimes resulting in excessive tissue injury and death.  Ref: 12874588 Surgery, 2003
- Furthermore, it was suggested that possible IL 18  [ **IL18** ]  IFN gamma  [ **IFNG** ]  induction of local proliferation of macrophages and CD8 cells might be an important mechanism for amplifying CD8 mediated macrophage dependent GN.  Ref: 12787406 Kidney Int, 2003
- Moreover, MHC class II DR expressing PMNs were also proved to express a high affinity receptor for immunoglobulin E IgE FcepsilonRI and to produce tumour necrosis factor alpha, interferon gamma  [ **IFNG** ]  and interleukin 18  [ **IL18** ]  following a challenge with an anti MHC class II DR monoclonal Ab MoAb or anti DR antiserum.  Ref: 12791081 Transfus Med, 2003
- We examined the effect of PGE2 on the expression of ICAM 1 and B7 in the human mixed leukocyte reaction MLR in the presence or absence of IL 18  [ **IL18** ] .METHODS We measured the expression of ICAM 1, B7.1, and B7.2 on human monocytes by flow cytometry and determined the associated production of interferon gamma  [ **IFNG** ]  and IL 12 by enzyme linked immunosorbent assay.  Ref: 12829919 Transplantation, 2003
- Deformed cortical bone and decreased turnover rate of lumbar trabecular bone are consistent with increased expression of IFN gamma  [ **IFNG** ]  and IL 18  [ **IL18** ]  in the bone marrow.  Ref: 12817749 J Bone Miner Res, 2003
- This study shows that inactivated parapoxvirus ovis Orf virus, strain D1701 PPVO, induces an autoregulatory cytokine response that involves the upregulation of IL 12, IL 18  [ **IL18** ] , IFN gamma  [ **IFNG** ]  and other T helper 1 type cytokines and their subsequent downregulation, which is accompanied by induction of IL 4.  Ref: 12810878 J Gen Virol< MedlineTA>J Gen Virol, 2003
- IFN gamma  [ **IFNG** ]  and IL 18  [ **IL18** ]  expression were determined in anti glomerular basement membrane GBM antibody Ab induced GN.  Ref: 12787406 Kidney Int, 2003
- Although a weak correlation was observed between IL 18  [ **IL18** ]  and IFN gamma  [ **IFNG** ]  concentrations, IFN gamma  [ **IFNG** ]  concentrations did not show any association with patients clinical parameters.  Ref: 12781884 Am J Surg, 2003
- BACKGROUND Interleukin 18  [ **IL18** ]  IL 18  [ **IL18** ] , a potent inducer of interferon gamma  [ **IFNG** ]  IFN gamma  [ **IFNG** ]  production, is a cytokine involved in the cell mediated immune response that is expressed during inflammatory and pathologic conditions.  Ref: 12787406 Kidney Int, 2003
- Although IL 12 is also an IFN gamma  [ **IFNG** ]  inducing factor, the activity of IL 18  [ **IL18** ]  but not IL 12 in models of sepsis and death is dependent on the intracellular cysteine protease IL 1beta converting enzyme caspase 1.  Ref: 12792854 J Infect Dis, 2003
- In this study, the authors observed that combined use of IL 18  [ **IL18** ]  and low dose IL 2 synergistically promoted in vitro proliferation of natural killer cells with up regulation of IL 2 receptor alpha and also synergistically stimulated cytolytic activity and interferon gamma  [ **IFNG** ]  production by these cells.  Ref: 12806277 J Immunother< MedlineTA>J Immunother, 2003
- Hepatic inflammatory activity was found to be associated more closely with IFN gamma  [ **IFNG** ]  than with IP 10 or IL 18  [ **IL18** ]  mRNA expression.  Ref: 12794718 J Med Virol, 2003
- ... IFN gamma  [ **IFNG** ] , IFNAR 1, STAT1alpha IRF 1, and IL 18  [ **IL18** ]  mRNA were upregulated.  Ref: 12794720 J Med Virol< MedlineTA>J Med Virol, 2003
- However, the capacity of IL 18  [ **IL18** ]  to produce IFN gamma  [ **IFNG** ]  may be altered depending on the severity of peritonitis.  Ref: 12781884 Am J Surg, 2003
- Dysregulation of IFN gamma  [ **IFNG** ]  production resulting from reduced activity of IL 18  [ **IL18** ]  would explain one of the mechanisms of immunosuppression in patients with chronic renal failure.  Ref: 12784053 Blood Purif, 2003
- Our results showed that the two mutants induced significantly less amount of IFN gamma  [ **IFNG** ]  from PBMC 13%, 48% of wild type rhIL 18 for DeltaNC, S, respectively, and the activation of NF kappaB also lower than wild type rhIL 18 69.7%, 89.8% of wild type rhIL 18 respectively, indicating that the deleted or mutated amino acids might be important for IL 18  [ **IL18** ]  function.  Ref: 12766800 Sheng Wu Hua Xue Yu Sheng Wu Wu Li Xue Bao Shanghai , 2003
- Because of the importance of IL 18  [ **IL18** ]  and IFN gamma  [ **IFNG** ]  in the Th1 response, the biology of IL 18  [ **IL18** ]  and IL 18BP is reviewed here in the context of the immunosuppression of chronic renal failure.  Ref: 12784053 Blood Purif, 2003
- Responsiveness of CD4 T cells to the IFN gamma  [ **IFNG** ]  inducing cytokines IL 12 and IL 18  [ **IL18** ]  is generally thought to be acquired only after stimulation via the TCR.  Ref: 12811838 Eur J Immunol, 2003
- Recent reports demonstrated that P. acnes treatment promotes IL 12 and IL 18  [ **IL18** ]  synthesis in mice inducing IFN gamma  [ **IFNG** ]  release, enhancement of IgG2a switch and inhibition of Th2 cell expansion.  Ref: 12880687 Immunol Lett, 2003
- IL 21 in Synergy with IL 15 or IL 18  [ **IL18** ]  Enhances IFN gamma  [ **IFNG** ]  Production in Human NK and T Cells.  Ref: 12759422 J Immunol, 2003
- Thus, deformed cortical bone and a decreased turnover rate of lumbar trabecular bone are characteristic of IL 18  [ **IL18** ]  TG mice, and these features might be associated with the increased expression of IFN gamma  [ **IFNG** ]  and IL 18  [ **IL18** ]  in the bone marrow.  Ref: 12817749 J Bone Miner Res, 2003
- IL 18  [ **IL18** ]  was not able to induce the binding of STATs to IFN gamma  [ **IFNG** ]  gene regulatory sites.  Ref: 12759422 J Immunol, 2003
- IL 18  [ **IL18** ]  and IL 21 enhanced IL 15 induced IFN gamma  [ **IFNG** ]  gene expression.  Ref: 12759422 J Immunol, 2003
- The aim of this study was to investigate the clinical significance of peritoneal IL 18  [ **IL18** ]  concentrations in patients with peritonitis.We measured IL 18  [ **IL18** ] , IFN gamma  [ **IFNG** ] , and IL 10 concentrations in the peritoneal fluid of 28 patients undergoing laparotomy for peritonitis.  Ref: 12781884 Am J Surg, 2003
- Because IFN gamma  [ **IFNG** ]  plays a key pathogenic role in the development of murine immunoinflammatory diabetes induced by multiple low doses of streptozotocin STZ we investigated the effect of negating the actions of endogenous IL 18  [ **IL18** ]  in this model by administering recombinant IL 18  [ **IL18** ]  binding protein Fc IL 18  [ **IL18** ]  bp Fc.  Ref: 12884303 Eur J ImmunolEur J Immunol, 2003
- IL 12, IL 18  [ **IL18** ] , and IFN gamma  [ **IFNG** ]  levels in supernatants of the liver MNCs stimulated with LPS in CLP mice were significantly higher than those in sham mice 7 days after the procedure.  Ref: 12874588 Surgery, 2003
- Nasal lavage specimens were assayed for interferon gamma  [ **IFNG** ] , IL 4, IL 10 and IL 12 and the RSV load determined by quantitative PCR. mRNA was extracted from stimulated peripheral blood mononuclear cells and Interferon gamma  [ **IFNG** ] , IL 4, IL 12 and IL 18  [ **IL18** ]  mRNA levels determined by PCR.  Ref: 12773328 Am J Respir Crit Care Med< MedlineTA>Am J Respir Crit Care Med, 2003
- In chronic hepatitis C, but not in chronic hepatitis B nor in liver disorders unrelated to viral infections, IP 10 expression was strongly correlated with the amount of transcripts for IFN gamma  [ **IFNG** ]  and to the amount of transcripts for the constitutively expressed macrophage derived cytokine IL 18  [ **IL18** ] .  Ref: 12794718 J Med Virol, 2003
- In addition, the STAPHYLOCOCCUS EPIDERMIDIS induced production of IFN gamma  [ **IFNG** ]  and IL 18  [ **IL18** ]  was assessed in whole blood cultures of HD patients as well as in 9 healthy subjects.  Ref: 12784048 Blood Purif, 2003
- Furthermore, serum IL 12 and IL 18  [ **IL18** ]  levels and liver MNCs IL 12, IL 18  [ **IL18** ] , and IFN gamma  [ **IFNG** ]  production were significantly increased in CLP mice compared with sham mice after LPS challenge.  Ref: 12874588 Surgery, 2003
- Changes in the local expression of IL 1beta, IL 2, IL 4, IL 6, IL 8, IL 10, IL 12, IL 15, IL 18  [ **IL18** ] , TNF alpha, IFN gamma  [ **IFNG** ]  and TGF beta 2 genes in the uterine adnexa and endometrium were studied in women with pyoinflammatory adnexal diseases.  Ref: 12802428 Bull Exp Biol Med< MedlineTA>Bull Exp Biol Med, 2003
- uNK cells proliferate, produce cytokines interferon gamma  [ **IFNG** ]  IFN gamma  [ **IFNG** ]  and interleukin 18  [ **IL18** ]  IL 18  [ **IL18** ]  and IL 27, and terminally differentiate into granulated lymphocytes.  Ref: 12887272 Reproduction, 2003
- Whereas IL 18  [ **IL18** ]  significantly induced the expression of ICAM 1, B7.1, and B7.2 on monocytes in MLR and the production of interferon gamma  [ **IFNG** ]  and IL 12, PGE2 inhibited these IL 18  [ **IL18** ]  initiated enhancements.  Ref: 12829919 Transplantation, 2003
